# Pulmonary GvHD: is it time to change the NIH diagnostic criteria?

**DOI:** 10.46989/001c.124551

**Published:** 2024-10-19

**Authors:** Martina Canichella, Poalo de Fabritiis

**Affiliations:** 1 Hematology St. Eugenio Hospital; 2 Policlinico Tor Vergata

**Keywords:** pulmonary graft-versus-host disease, allogeneic stem cell transplantation, Bronchiolitis obliterans syndrome

Late-onset, non-infectious pulmonary complications of hematopoietic stem cell transplantation (HSCT) can manifest with different patterns (obstructive, restrictive, and mixed). However, bronchiolitis obliterans syndrome (BOS) is the only entity of pulmonary chronic graft-versus-host disease (cGvHD) formally recognized by the National Institutes of Health (NIH).^1^ BOS is characterized by changes in pulmonary function detected by spirometry, while the histopathological pattern is the bronchiolitis obliterans (BO), characterized by progressive airflow obstruction due to subepithelial inflammatory and fibrotic narrowing of the bronchioles. BOS affects approximately 14% of patients with cGvHD and is associated with high mortality and a marked reduction in quality of life.^2^

However, the incidence of BOS may be underestimated due to its insidious clinical presentation with nonspecific symptoms such as mild dyspnea, a dry and non-productive cough, and a wheezing that decreases exercise tolerance. The final stage is marked by oxygen dependency with a high risk of developing infectious complications.

Several risk factors have been associated with the development of BOS which can be distinguished as patient-related and transplant-related. With regards to patient’s history, a high-risk disease, a reduction of pretrasplant forced expiratory volume in 1 second (FEV1) and, mostly, a pretrasplant lung disease are associated with a high-risk of developing BOS. On the other hand, negative factors related to the transplant strategy such as the use of busulfan (even administered at lower dose), unrelated and female donor, CMV seropositivity; on the contrary, the use of anti-thymocyte globulin (ATG) in the GvHD prevention, was found to have a protective role.^3^

In general, the type of GvHD prophylaxis is crucial for BOS onset. In particular, several studies in the setting of sibling or matched unrelated donor (MUD) HSCT have shown that the reduction of cGvHD is influenced by the use of ATG. In 2022 Luznik et al. compared the incidence of cGvHD in a randomized study using three different types of prophylaxis: *ex vivo* selective T-cell depletion, post-trasplant cyclophosphamide (PTCY), and tacrolimus plus methotrexate (TC-MTX). The incidence of moderate-severe cGvHD was lower for the first group, and higher for patients treated with PTCY and TC-MTX.^4^ Moreover, Bacigalupo et al. demonstrated that patients who did not receive ATG showed a significant reduction in lung function during follow-up, compared to those who did receive it.^5^

Recently, with the wider use of haploidentical peripheral blood hematopoietic cell transplantation (haplo-HCT) with PTCY as GvHD prophylaxis, there has been growing attention to the development of novel strategies to reduce the risk of cGvHD. In this regard, a recent study demonstrated that adding ATG to the PTCY regimen significantly reduces the incidence of cGvHD.^6^

Taken together, in order to reduce the risk of BOS onset, the planning of conditioning regimens should avoid, if possible, the use of busulfan. Indeed, in the follow-up, close monitoring with pulmonary functional tests (PFT) should be done to patients who present multiple risk factors, especially those who have not received ATG.

Treating BOS remains challenging, primarily because it is often diagnosed in advanced stages. The strategy includes macrolides, immunosuppression and extracorporeal photoaferesis (ECP).

The choice of treatment must be carefully considered. In particular, the association based on the fluticasone-azithromycin-montelukast (FAM) must be used only in the case of a confirmed diagnosis of BOS,^7^ being azithromycin correlated with an incidence of high risk of relapse.^8^

However, if signs of cGvHD are present in other organs, a systemic therapy tailored to the patient should be preferred to the FAM.

Recently, new agents with different pathways of action have been experimented in the treatment of cGvHD with promising results also in BOS. Among these, ruxolitinib, a selective JAK 1/2 inhibitor, is the standard second-line treatment of acute and chronic GvHD.^9,10^ With regard to pulmonary manifestation, ruxolitinib was demonstrated to reduce symptoms - measured with the Lee scale - improving quality of life and allowing reduction of the corticosteroid dose. Side effects, mostly the risk of infections, have to be considered when ruxolitinib is used. Another effective compound in the treatment of cGvHD is belumosudil, an oral selective inhibitor of rho-associated coiled-coil-containing protein kinase-2 (ROCK2) demonstrating activity also in pulmonary cGvHD.^11^

Finally, axatilimab, a monoclonal antibody recognizing the ligand-binding domain on colony-stimulating factor 1 receptor (CSF-1R) showed a safety profile and effectiveness in a heavily pretreated patient, making it a candidate for rapid entry into the therapeutic algorithm for cGvHD.^12^

In 2014, the NIH revised the criteria for diagnosing and assessing the severity of cGvHD.^1^ The document refined the previous 2005 consensus, and addressed certain areas of controversy or confusion, such as the overlap of cGvHD subcategories. A turning point for pulmonary cGvHD was the diagnostic shift from the lung biopsy to spirometry, as a surrogate of this obstructive syndrome. A lung biopsy was not feasible in a high proportion of patients due to a high risk of complications (bleeding and infections), and it contributed to an underestimate of the diagnosis, potentially resulting in unchecked progression to an advanced stage of the disease. The NIH criteria emphasize spirometry parameters together with clinical and radiographic features while, simultaneously, ruling out an infectious cause for any decline in lung function. The same document modified the lung scoring, considering only FEV1 and not diffusion lung carbon monoxide (DLCO). This simplified score assessment increased the specificity for obstructive pattern. These criteria, however, have some critical aspects: the spirometry-based diagnosis and monitoring strategies do not permit the early identification of BOS, which is crucial to initiate treatment promptly and improve outcomes. Furthermore, these criteria do not adequately address the non-BOS manifestations of lung cGvHD, which are common and significantly impact quality of life.

Spirometry is a poor sensitivity test for detecting peripheral airway obstruction as demonstrated in animal models.^13^ So, when spirometry abnormalities are detected, the BOS process is already well advanced. Indeed, a threshold value of FEV1 lower than 75% indicates an advanced degree of obstruction, and the FEV1 is not strictly correlated with a contemporary decline in vital capacity, unlike a ratio of 0.7, as required by another NIH diagnostic criterion. The limitations in early detection and monitoring of BOS triggered an investigation into the potential role of different spirometry parameters and PFT. In 2020, a panel of experts issued NIH guidelines for the early diagnosis of cGvHD, including pulmonary manifestation.^14^ They took into consideration the categories of pre-BOS and BOS-stage 0p, the latter being a stage used in lung allograft recipients to identify airflow obstruction prior to the development of symptoms. It is defined by the 10% decline in FEV1 and/or 25% decline in FEF_25-75%_ (forced expiratory flow) compared to pre-HSCT PFT parameters.^15^ In the setting of HSCT, this stage has been demonstrated to predict the onset of BOS with high negative predictive value, and the application of PFT should result in a more sensitive and feasible detection of peripheral airway changes. A growing interest was about the multiple breath washout (MBW) and oscillometry to detect incipient obstructive alteration of airways.^16^ The MBW assesses gas mixing, which is predominant in peripheral airways, representing about 95% of lung volume. Through the lung clearance index (LCI), this test is able to detect heterogeneous peripheral airways alterations, characteristic of the BOS process. MBW also efficiently detects peripheral disfunction in several lung conditions such as cystic fibrosis. Oscillometry is another breathing test also applicable at pediatric ages. It measures lung impedance through the respiratory system resistance (Rrs) and reactance (Xrs). A recent review on the use of MBW and oscillometry in allografted patients revealed that MBW is always abnormal prior to HSCT but was more frequently abnormal than spirometry in patients with pulmonary cGvHD. Oscillometry indices were often normal at baseline and frequently abnormal in those who developed pulmonary cGvHD. MBW and oscillometry hold promise for early identification of peripheral airway dysfunction in HSCT recipients.^16^

Another critical aspect of NIH in the diagnosis of BOS is the exclusion of respiratory infections. This criterion does not consider the possible association between infection and cGvHD, inducing the clinician to attribute the decline of the pulmonary function to the infection, instead of the cGvHD. As mentioned above, the NIH criteria have certainly facilitated the diagnosis of BOS, but have overlooked several restrictive patterns of cGvHD, such as cryptogenic organizing pneumoniae (COP) and interstitial forms, which are often characterized by a reduction in the DLCO. These distinct forms are relatively common (occurring in 16-20% of cases) and have a significant negative impact on overall survival.^17^ It is therefore crucial to identify and treat them appropriately. It is important to note, however, that not all cases of restrictive lung disease are directly associated with pulmonary cGvHD; they may also manifest as part of cGvHD affecting other organs, such as scleroderma or fibrosis of the visceral pleura. To identify those cases with non-BOS pulmonary cGVHD, Pang and colleagues applied the International Society for Heart and Lung Transplantation (ISHLT) diagnostic criteria in 166 allograft patients with pulmonary cGvHD.^18^ The rationale behind this study was based on the shared pathogenesis and clinical manifestations of BOS and Chronic Lung Allograft Dysfunction. Their findings revealed that half of the patients diagnosed with lung cGVHD would not have been identified using the current NIH criteria.

Recently, in order to improve the early identification of BOS, prognostic biomarkers have been related to BOS onset. Murine models, subsequently confirmed in humans, have demonstrated that cell actors in the pathogenesis of bronchiolar obstruction are represented by macrophages and B lymphocytes, particularly the CD19-CD21 low subpopulation of B-cells. The cytokines and factors responsible for this pathogenesis are represented by transforming growth factor-B (TGFβ) and B-cell activator factors (BAF). High levels of these two biomarkers have been correlated with poor outcome.^19^ However, there are currently no validated markers that can be applied in clinical practice.

In conclusion, the strict application of NIH criteria is unsatisfactory in early diagnosis of BOS and recognition of the non-BOS form of pulmonary cGvHD. In the planning of HSCT, the identification of those patients at risk to develop cGvHD and pulmonary cGvHD is crucial to ameliorate the quality of life of long-term survivors. The close monitoring with more sensitive PFT validated by further studies will be fundamental for early diagnosis and for a pre-emptive approach to avoid the establishment of a more advanced stage of the disease (Figure 1).

**FIGURE 1.**
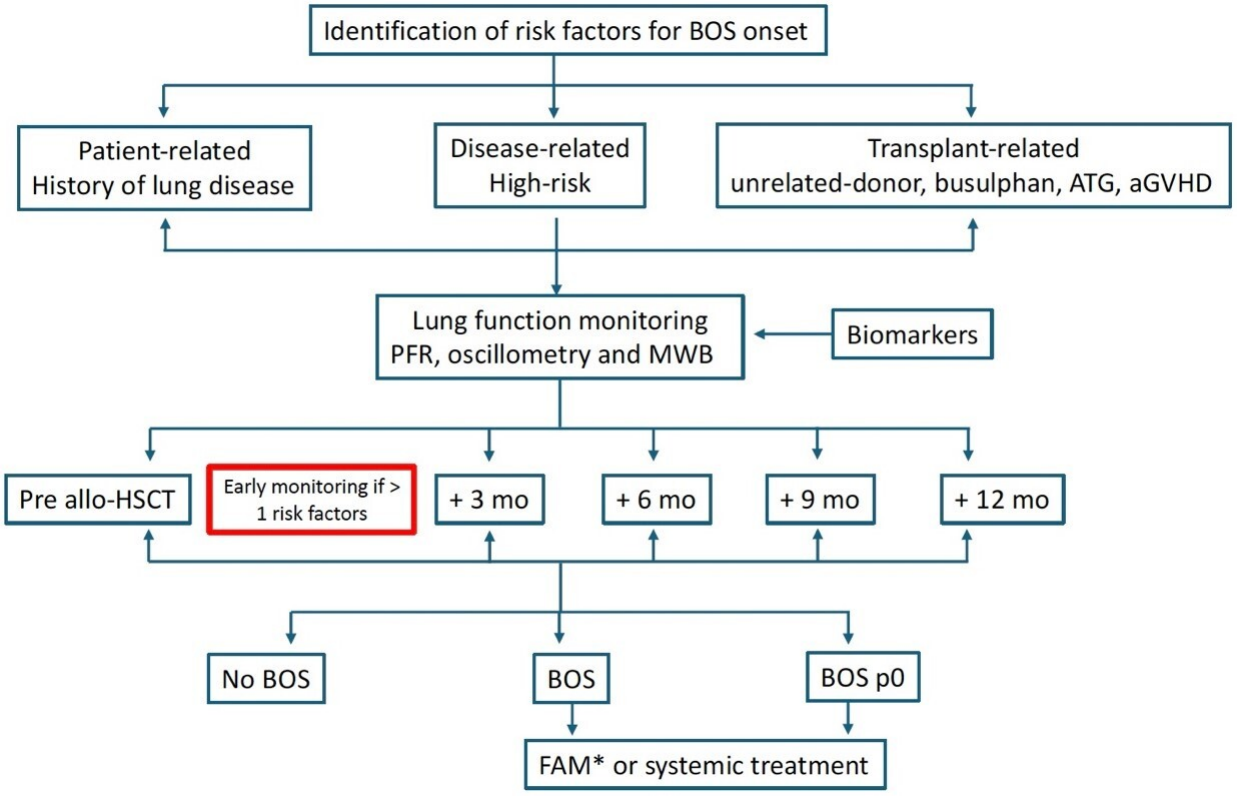
Proposed diagnostic algorithm for BOS diagnosis. BOS bronchiolitis obliterans syndrome; ATG anti-thymocyte globulin; GvHD: graft-versus-host disease; MWB multiple breath washout; mo: months; FAM Fluticasone-azithromycin-montelukast *FAM should be administered only with cartain diagnosis of BOS.

## AUTHOR CONTRIBUTIONS

Martina Canichella designed the research and wrote the paper. Paolo de Fabritiis reviewed and wrote the paper.

## CONFLICT OF INTEREST STATEMENT

The authors declare no conflict of interest.

## References

[ref-368354] Jagasia M. H., Greinix H. T., Arora M., Williams K. M. (2015). National Institutes of Health Consensus Development Project on Criteria for Clinical Trials in Chronic Graft-versus-Host Disease: I. The 2014 Diagnosis and Staging Working Group report. Biol Blood Marrow Transplant.

[ref-368355] Socié G., Stone J. V., Wingard J. R. (1999). Late Effects Working Committee of the International Bone Marrow Transplant Registry. Long-term survival and late deaths after allogeneic bone marrow transplantation. N Engl J Med.

[ref-368356] Gazourian L., Rogers A. J., Ibanga R. (2014). Factors associated with bronchiolitis obliterans syndrome and chronic graft-versus-host disease after allogeneic hematopoietic cell transplantation. Am J Hematol.

[ref-368357] Luznik L., Pasquini M. C., Logan B. (2022). Randomized Phase III BMT CTN Trial of Calcineurin Inhibitor-Free Chronic Graft-Versus-Host Disease Interventions in Myeloablative Hematopoietic Cell Transplantation for Hematologic Malignancies. J Clin Oncol.

[ref-368358] Bacigalupo A., Lamparelli T., Barisione G. l. (2006). Thymoglobulin prevents chronic graft-versus-host disease, chronic lung dysfunction, and late transplant-related mortality: long-term follow-up of a randomized trial in patients undergoing unrelated donor transplantation. Biol Blood Marrow Transplant.

[ref-368359] Battipaglia G., Labopin M., Blaise D. (2022). Impact of the Addition of Antithymocyte Globulin to Post-Transplantation Cyclophosphamide in Haploidentical Transplantation with Peripheral Blood Compared to Post-Transplantation Cyclophosphamide Alone in Acute Myelogenous Leukemia: A Retrospective Study on Behalf of the Acute Leukemia Working Party of the European Society for Blood and Marrow Transplantation. Transplant Cell Ther.

[ref-368360] Williams K. M., Cheng G. S., Pusic I. (2016). Fluticasone, Azithromycin, and Montelukast Treatment for New-Onset Bronchiolitis Obliterans Syndrome after Hematopoietic Cell Transplantation. Biol Blood Marrow Transplant.

[ref-368361] Vallet N., Le Grand S., Bondeelle L. (2022). Azithromycin promotes relapse by disrupting immune and metabolic networks after allogeneic stem cell transplantation. Blood.

[ref-368362] Zeiser R., Polverelli N., Ram R. (2021). Ruxolitinib for Glucocorticoid-Refractory Chronic Graft-versus-Host Disease. N Engl J Med..

[ref-368363] Penack O., Marchetti M., Aljurf M. (2024). Prophylaxis and management of graft-versus-host disease after stem-cell transplantation for haematological malignancies: updated consensus recommendations of the European Society for Blood and Marrow Transplantation. Lancet Haematol.

[ref-368364] Cutler C., Lee S. J., Arai S. (2021). Belumosudil for chronic graft-versus-host disease after 2 or more prior lines of therapy: the Rockstar Study. Blood.

[ref-368365] Kitko C. L., Arora M., DeFilipp Z. (2023). Axatilimab for Chronic Graft-Versus-Host Disease After Failure of at Least Two Prior Systemic Therapies: Results of a Phase I/II Study. J Clin Oncol.

[ref-368366] Brown R., Woolcock A. J., Vincent N. J. (1969). Physiological effects of experimental airway obstruction with beads. J Appl Physiol.

[ref-368367] Kitko C. L., Pidala J., Schoemans H. M. (2021). National Institutes of Health Consensus Development Project on Criteria for Clinical Trials in Chronic Graft-versus-Host Disease: IIa. The 2020 Clinical Implementation and Early Diagnosis Working Group Report. Transplant Cell Ther..

[ref-368368] Abedin S., Yanik G. A., Braun T. (2015). Predictive value of bronchiolitis obliterans syndrome stage 0p in chronic graft-versus-host disease of the lung. Biol Blood Marrow Transplant.

[ref-368369] Sonneveld N., Rayment J. H., Usemann J. (2023). Multiple breath washout and oscillometry after allogenic HSCT: a scoping review. Eur Respir Rev.

[ref-368370] Afessa B., Litzow M., Tefferi A. (2001). Bronchiolitis obliterans and other late onset non-infectious pulmonary complications in hematopoietic stem cell transplantation. Bone Marrow Transplantation.

[ref-368371] Pang Y., Charya A. V., Keller M. B. (2022). The ISHLT chronic lung allograft dysfunction consensus criteria are applicable to pulmonary chronic graft-versus-host disease. Blood Adv.

[ref-368372] MacDonald K. P., Blazar B. R., Hill G. R. (2017). Cytokine mediators of chronic graft-versus-host disease. J Clin Invest.

